# Ethanol deprivation and central 5-HT deficiency differentially affect the mRNA editing of the 5-HT_2C_ receptor in the mouse brain

**DOI:** 10.1007/s43440-023-00545-6

**Published:** 2023-11-03

**Authors:** Magdalena Zaniewska, Natalia Alenina, Sebastian Fröhler, Wei Chen, Michael Bader

**Affiliations:** 1 Department of Pharmacology, Laboratory of Pharmacology and Brain Biostructure, Maj Institute of Pharmacology, Polish Academy of Sciences, 12 Smętna Street, 31-343 Kraków, Poland; 2 Max-Delbrück-Center for Molecular Medicine, Robert-Rössle-Str. 10, 13125 Berlin, Germany; 3 DZHK (German Center for Cardiovascular Research), Partner Site Berlin, Berlin, Germany; 4 Laboratory for New Sequencing Technology, Max-Delbrück-Center for Molecular Medicine, Berlin Institute for Medical Systems Biology, Robert-Rössle-Str. 10, 13125 Berlin, Germany; 5 Charité Universitätsmedizin Berlin, Corporate Member of Freie Universität Berlin, Berlin, Germany; 6 Institute for Biology, University of Lübeck, Lübeck, Germany; 7 Present Address: Department of Systems Biology, School of Life Science, Southern University of Science and Technology, Shenzhen, 518055 China

**Keywords:** Deep sequencing, Ethanol cessation, 5-HT_2C_ receptor mRNA editing, Mice, *Tph2* knockout, *Tph2* transcript level

## Abstract

**Background:**

Serotonin (5-HT) 5-HT_2C_ receptor mRNA editing (at five sites, A–E), implicated in neuropsychiatric disorders, including clinical depression, remains unexplored during alcohol abstinence—often accompanied by depressive symptoms.

**Methods:**

We used deep sequencing to investigate 5-HT_2C_ receptor editing in mice during early ethanol deprivation following prolonged alcohol exposure and mice lacking tryptophan hydroxylase (TPH)2, a key enzyme in central 5-HT production. We also examined *Tph2* expression in ethanol-deprived animals using quantitative real-time PCR (qPCR).

**Results:**

Cessation from chronic 10% ethanol exposure in a two-bottle choice paradigm enhanced immobility time and decreased latency in the forced swim test (FST), indicating a depression-like phenotype. In the hippocampus, ethanol-deprived “high ethanol-drinking” mice displayed reduced *Tph2* expression, elevated 5-HT_2C_ receptor editing efficiency, and decreased frequency of the D mRNA variant, encoding the less-edited INV protein isoform. *Tph2*^–/–^ mice showed attenuated receptor editing in the hippocampus and elevated frequency of non-edited None and D variants. In the prefrontal cortex, *Tph2* deficiency increased receptor mRNA editing at site D and reduced the frequency of AB transcript, predicting a reduction in the corresponding partially edited VNI isoform.

**Conclusions:**

Our findings reveal differential effects of 5-HT depletion and ethanol cessation on 5-HT_2C_ receptor editing. Central 5-HT depletion attenuated editing in the prefrontal cortex and the hippocampus, whereas ethanol deprivation, coinciding with reduced *Tph2* expression in the hippocampus, enhanced receptor editing efficiency specifically in this brain region. This study highlights the interplay between 5-HT synthesis, ethanol cessation, and 5-HT_2C_ receptor editing, providing potential mechanism underlying increased ethanol consumption and deprivation.

**Graphical abstract:**

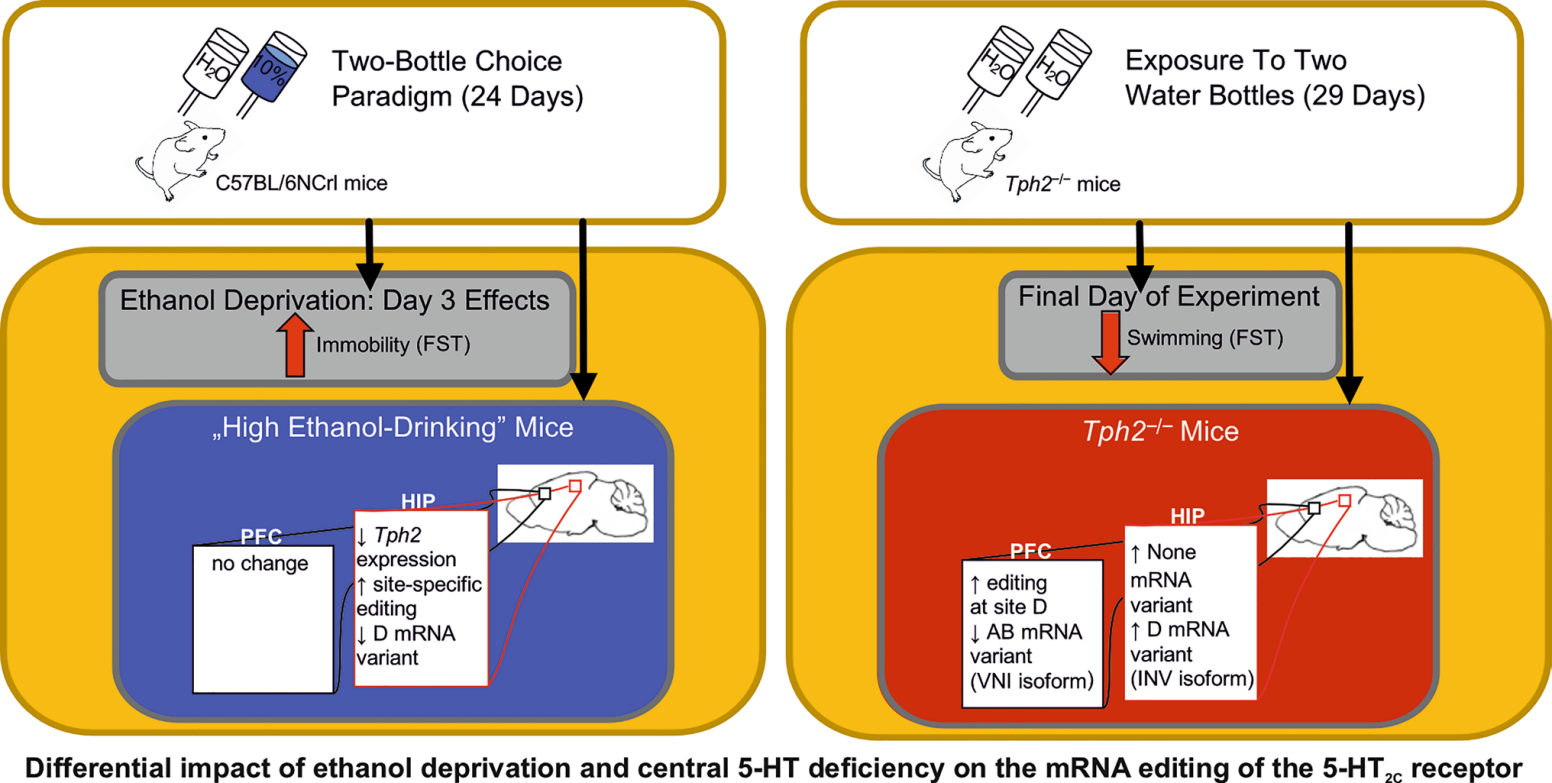

**Supplementary Information:**

The online version contains supplementary material available at 10.1007/s43440-023-00545-6.

## Introduction

Serotonin (5-HT) is a key neurotransmitter in the brain, synthesized within serotonergic neurons from tryptophan by tryptophan hydroxylase 2 (TPH2; distinct from peripheral 5-HT production, which involves TPH1) [1]. Upon neuronal activation, 5-HT is released into the synaptic cleft, where it interacts with various receptors (14 receptor subtypes). It is then reuptaken into serotonergic neurons by the 5-HT transporter and either packed back to vesicles or degraded by monoamine oxidase A. The 5-HT-synthesizing neurons, located in the brainstem raphe nuclei, have extensive projections that innervate all regions of the brain and the spinal cord. Its broad distribution in the brain makes 5-HT an important neurotransmitter regulating many physiological conditions, including aggression, mood, appetite, and learning and memory processes [2].

Numerous neuropsychiatric disorders, including ethanol dependence, have been associated with the disruption of 5-HT homeostasis in the brain [2–5] and an important role concerning the interaction with ethanol has been attributed to 5-HT_2C_ receptors [6–9]. In clinical research, certain groups of alcoholics who have ceased alcohol drinking have shown attenuated 5-HT function, as evidenced by lower concentrations of its metabolite 5-hydroxyindoleacetic acid (5-HIAA) in the cerebrospinal fluid [10, 11]. In preclinical studies, chronic ethanol was associated with alterations within the 5-HT system (e.g., TPH2 activity and expression, 5-HIAA levels, 5-HT turnover, and 5-HT_1A_ receptor sensitivity) in the raphe nuclei, cortex, hippocampus, and/or striatum [12, 13]. Recently, we have shown a decrease in the transcript level of *Tph2* in the prefrontal cortex of mice exhibiting elevated ethanol consumption [5], suggesting heightened sensitivity of 5-HT transmission in “high ethanol-drinking” animals compared to low ethanol drinkers. Ethanol cessation also induced changes in serotonergic homeostasis, i.e., reduced 5-HT levels, 5-HT/tryptophan and/or 5-HT/5-HIAA ratios in the hippocampus, cortex and/or amygdala [14, 15]. Importantly, attenuated 5-HT signaling has been linked to depressive symptoms [16], and these, in turn, are frequently observed in individuals struggling with alcohol addiction [17]. The co-occurring depression in alcoholics is associated with greater likelihood of relapse and a worse prognosis [18].

Reducing tryptophan in alcoholic individuals had no impact on drinking and drug craving evoked by environmental stimuli [19–21], however, it did induce depression in detoxified patients [21]. In turn, selective serotonin reuptake inhibitors (SSRIs) reduced alcohol consumption in alcoholic subjects [22, 23], although other reports did not confirm this observation [24, 25]. Preclinical studies on the impact of SSRIs on ethanol drinking in rats have also provided heterogenous results, with studies reporting either a decrease or an escalation in ethanol consumption following SSRI treatment [26–28]. These discrepancies may be due to the heterogeneity of alcohol addicts, emphasizing the necessity for additional research on the molecular mechanisms underlying the co-occurrence of alcohol use disorder and depression.

Imbalance in 5-HT homeostasis may influence the 5-HT_2C_ receptor mRNA editing process [29–31]—a post-transcriptional modification, resulting in the conversion of adenosine-to-inosine (A-to-I) by adenosine deaminases acting on RNA (ADARs) [32]). The receptor’s coding sequence for the second intracellular loop contains five RNA editing sites (labeled A to E). By converting one or more editing sites to inosine, which is recognized as guanosine during translation, the sequence of three amino acid residues (I156, N158, and I160) in the non-edited receptor (INI) may be modified. This process could theoretically generate 24 receptor protein isoforms. Some of these isoforms exhibit reduced protein function in vitro, including constitutive receptor activity, agonist binding affinity and potency [32–36].

Notably, in vivo studies have shown that a sole expression of the highly functional INI isoform of the 5-HT_2C_ receptor dampened the enhanced intake of ethanol in C57BL/6 J mice [37, 38] with a high preference for ethanol [39], while the exclusive expression of the fully-edited (VGV) receptor isoform in C57BL/6 J mice had no impact on ethanol drinking behavior [40]. In the case of depressive behaviors, mice expressing the INI isoform displayed behavior indicative of depression (evidenced as an increased duration of immobility) in the forced swim test (FST), whereas VGV mice showed an antidepressant phenotype in the FST (indicated as reduced immobility time) and tail suspension test (with decreased time spent immobile) [41, 42]. Overall, the findings obtained in INI and VGV mice suggest that the intensity of the 5-HT_2C_ receptor editing process modulates ethanol drinking and depression-like behaviors.

To date, the 5-HT_2C_ receptor mRNA editing in alcohol addicts has not yet been investigated. Changes in receptor editing in the brain, particularly the prefrontal cortex, have been found in individuals diagnosed with major depressive disorder and/or those who died by suicide [35, 43–46], although not consistently across all patient groups [35, 46, 47]. Preclinical investigations by Watanabe et al. [38] revealed that exposure to ethanol vapor enhanced 5-HT_2C_ receptor mRNA editing in the dorsal raphe nuclei and nucleus accumbens of mice. However, the effects of ethanol cessation on this molecular process have not been explored so far.

Taking into account the aforementioned considerations, we conducted a study to explore the relationship between 5-HT synthesis in the brain and 5-HT_2C_ receptor RNA editing during ethanol cessation. We hypothesized that the expression of *Tph2* and mRNA editing of 5-HT_2C_ receptor might be altered in “high ethanol-drinking” mice following ethanol deprivation. We also evaluated the receptor editing pattern in *Tph2-*deficient (*Tph2*^*−/−*^) mice, which lack central 5-HT entirely [48]. Our previous research revealed that these knockout mice show abnormal behavior in the FST and higher ethanol consumption compared to wild-type counterparts [5, 49], indicating an association between central 5-HT, depression-like behaviors, and ethanol drinking in mice. In this research, we utilized a two-bottle drinking paradigm to model ethanol intake. C57BL/6N mice were exposed to chronic ethanol consumption and subsequently underwent spontaneous cessation. Depression-like behavior was assessed during the early stage of ethanol deprivation (day 3) in the FST. The level of *Tph2* transcript in the brain of “high ethanol-drinking” animals deprived of ethanol was evaluated using quantitative real-time PCR (qPCR). Deep sequencing approach, known for its accuracy in assessing the mRNA editing of 5-HT_2C_ receptor in the rodent brain [50], was employed to analyze the receptor RNA editing profile in ethanol-deprived high-ethanol drinkers and *Tph2*^*−/−*^ mice. The alterations at the molecular level were examined in both the entire hippocampus and prefrontal cortex, regions of the brain linked to alcohol addiction and depression, and known to be sensitive to 5-HT perturbations [3, 51, 52].

## Materials and methods

### Animals

Animal experimental procedures were in compliance with the European Community Council Directive 2010/63/EU for animal experiments, and received approval from the local animal welfare and ethical review body (State Office for Health and Social Affairs Berlin − LAGeSo) under the reference G 0343/09, with the approval date 24 March 2010.

Animals were housed as described before [5], in individually ventilated cages (Tecniplast Deutschland, Hohenpeissenberg, Germany) in a colony room maintained at 45 ± 5% humidity. Bedding and paper nesting material for the animals were provided in each cage. The behavioral analyses were performed within the light phase of the light-dark cycle (8:00 a.m. to 4:00 p.m.).

**Fig. 1:**
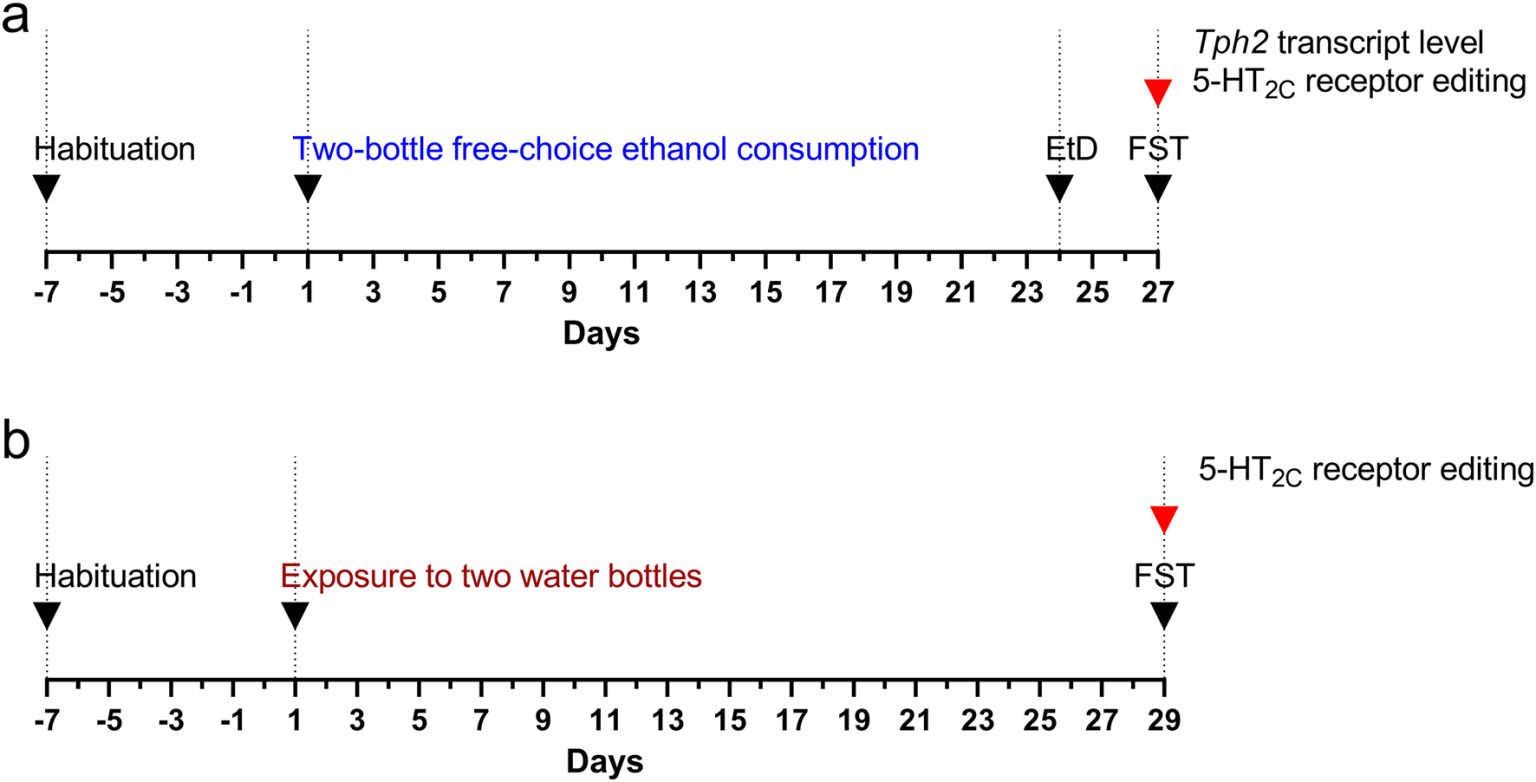
Experimental timeline. **a** Analysis of *Tph2* expression and editing of 5-HT_2C_ receptor in C57BL/6N mice deprived of ethanol. Individually housed mice were first habituated to two bottles and were then given access to water and 10% ethanol (a two-bottle free-choice test) for a period of 24 days. Next, animals underwent an ethanol deprivation period (EtD). On the third day of ethanol cessation, the phenotype of mice was assessed in the forced swim test (FST). After the FST, mice were sacrificed (red arrow), and the *Tph2* expression and 5-HT_2C_ receptor mRNA editing were evaluated; **b** The assessment of the 5-HT_2C_ receptor mRNA editing in *Tph2*^*−/−*^ mice. Individually housed *Tph2*^*−/−*^ and *Tph2*^+*/*+^ mice were first habituated to two water bottles. After 29 days, the phenotype of the mice was determined in the FST. After the test, mice were sacrificed (red arrow), and the 5-HT_2C_ receptor editing was analyzed

In the ethanol drinking paradigm (Fig. 1a), male C57BL/6N mice (*n* = 16; Charles River, Sulzfeld, Germany; C57BL/6NCrl) with initial weights of 26.56±0.52 g (ca. 14 weeks old) were utilized.

Male *Tph2*^*−/−*^ mice on a C57BL/6NCrl background (*n* = 4) and wild-type mice (*Tph2*^+*/*+^; *n* = 4) with initial weights of 23.54 ± 1.22 g and 25.41 ± 0.34 g, respectively, and aged approximately 10 weeks were employed in the study. Generation and genotyping of C57BL/6 *Tph2*^+*/*+^ and *Tph2*^*−/−*^ mice were previously outlined [5]. Randomly selected *Tph2*^+*/*+^ and *Tph2*^*−/−*^ mice in the present investigation, served as control animals in the behavioral analyses outlined in detail in our earlier investigation [5]. Similar to the housing conditions of the control mice subjected to a two-bottle free-choice procedure in the current study (water-drinking group), the *Tph2*^+*/*+^ and *Tph2*^*−/−*^ mice were individually housed with access to two water bottles for ca. four weeks (29 days). On the final day of the experiment, the mice underwent the FST, and the same parameters as those applied to the ethanol-deprived animals were evaluated. The brain tissue from these animal groups was used to assess the 5-HT_2C_ receptor mRNA editing profile (Fig. 1b).

### Behavioral studies

#### Drugs

Ethanol (10%; *v/v*) was diluted using 96% ethyl alcohol (Merck, Darmstadt, Germany) and tap water.

#### Ethanol drinking procedure

Individually housed mice (*n* = 16) were habituated to two bottles (Fig. 1a), as described before [5]. Animals underwent a two-bottle free-choice test within their home cages, based on our earlier study [5] with small modifications. Briefly, randomly selected animals (*n* = 8) were provided with water and 10% ethanol [53–55] for 24 days (Fig. 1a). Control animals (*n* = 8) had access to two water bottles throughout the study. The bottles (water and ethanol) were weighed at 24-h intervals. Daily ethanol consumption was determined as g of pure ethanol ingested within 1 day per kg of body weight (g/kg/day) [53]. Preference was assessed by measuring g of ethanol consumed per day as the percentage of total liquid consumed (%). Liquid intake was calculated as ml of liquid (water or water + ethanol) drunk within 1 day per kg of body weight (ml/kg/day). We also determined cumulative ethanol consumption (g of pure ethanol ingested per kg of body weight over 24 days, g/kg/24 days) and average intake of ethanol (g of pure ethanol consumed within 24 days per kg of body weight per day, g/kg/day).

#### Evaluation of animal’s behavior in the forced swim test (FST)

After 24 days of ethanol exposure, mice underwent a 3-days deprivation period, during which the animals had unrestricted access to a water bottle and remained in their home cages. On the third day, the behavior of animals was evaluated in the FST (Fig. 1a), following a previously described method [5]. The measured parameters were as follows: latency to the first immobility, duration of immobility, climbing, and swimming behaviors.

The specific period of ethanol deprivation was determined based on earlier observations in mice. This data indicated that the greatest depression-like phenotype (i.e., elevation in immobility behavior), was reported on this particular day of ethanol cessation (Zaniewska, Alenina, and Bader, unpublished observations).

#### Classification of animals as high and low-ethanol drinkers

Based on our previous investigation [5], which demonstrated differences in *Tph2* transcript levels between mice drinking high and low ethanol levels during the final week of ethanol consumption, we categorized the mice into high and low-ethanol drinkers using median split. Animal classification was outlined in detail in our previous study [5]. For both groups of ethanol drinkers, the average intake of ethanol (g/kg/day) and ethanol preference (%) were calculated based on data collected during the last week of access to ethanol. Additionally, the immobility time during the “test” (s) was computed. For molecular analyses (*Tph2* transcript level and 5-HT_2C_ receptor mRNA editing; Fig. 1a), we included brain tissue samples obtained from the “high ethanol-drinking” group (*n* = 4) and randomly selected water-drinking animals (*n* = 4).

### Molecular analyses

#### Preparation of brain tissue samples

Using the previously published method [5], after the FST, animals were sacrificed, and the hippocampi and prefrontal cortices were extracted. RNA was then isolated from these two brain regions.

#### Real-Time PCR

Further RNA cleanup and analysis of *Tph2* transcript level were performed according to the previously published study [5]. Data were normalized to the transcript level of TATA box binding protein (*Tbp*). A comparative cycle threshold (*C*_T_) method [56] was employed.

#### Deep sequencing

The RNA processing and analysis of mRNA editing of 5-HT_2C_ receptor, by deep sequencing technology, were conducted by applying previously outlined methods [50] with small modifications. Briefly, the 5-HT_2C_ receptor edited region was amplified by PCR with a PCR gradient thermal cycler (MJ Research PTC-200, MJ Research, Waltham, MA, USA), *Taq* DNA Polymerase (Invitrogen), and cDNA (100 ng/PCR reaction; four PCR reactions/each condition). The primers utilized for amplification were as follows (NM_008312.4): forward HT2C 51: 5′-xxxxxxTTTTCAACTGCGTC CATCATGCACCT-3′; reverse HT2C 3: 5′-ATCTTCATGATGGCCTTAGTCCG-3′ [50, 57]. To distinguish between the 31 conditions (4 treatment groups: water/ethanol, *Tph2*^+*/*+^*/Tph2*^*−/−*^; 2 brain regions: the hippocampus/prefrontal cortex; 3–4 animals/group), a unique six-nucleotide barcode for each condition (xxxxxx, e.g., AAGTCC, GTCCAA, TTGGTT) was incorporated at the 5′-terminus of the forward primer. As a control, the genomic DNA was used to amplify the non-edited region. After clean-up, 400 ng of barcoded PCR products were mixed and the created Illumina TruSeq DNA library containing 32 PCR amplicons was then sequenced on an Illumina HiSeq 2000 (San Diego, CA, USA).

The measured parameters were as follows: site-specific editing (%), frequency of 5-HT_2C_ receptor transcript variants (%), and frequency of protein isoforms (%). Site-specific editing was determined as the number of sequencing reads for G as the percentage of the total number of reads for A and G (G/(G+A)). The frequency of mRNA variants (or receptor isoforms) was determined as the number of sequencing reads for the appropriate variant (or receptor isoform) as the percentage of total number of sequencing reads.

### Statistical analyses

The data are presented as means (± SEM) or median with percentile range. The Shapiro–Wilk test was applied to assess the normality of data distribution. After confirming assumptions such as normal distribution and homogeneity of variance, suitable statistical tests were chosen. For two group comparisons, Student’s *t*-test for independent samples was utilized. If unequal variances were found, a Student’s *t*-test with Welch correction was used. When the data were not normally distributed, the Mann–Whitney *U* test was applied. Ethanol intake and preference data were processed by applying repeated measures analysis of variance (ANOVA). A one-way ANOVA with repeated measures was employed to examine the liquid intake data (factors: treatment (ethanol), day), as well as ethanol consumption and preference in high and low-ethanol drinkers (factors: ethanol group, day). A one-way ANOVA was applied to analyze the effect of ethanol deprivation on the immobility time in high and low-ethanol drinkers. A two-way ANOVA was conducted to assess the impact of the brain region (factors: brain region, editing site), treatment (factors: ethanol cessation, editing site), or genotype (factors: genotype, editing site) on site-specific editing, and, additionally, the effect of treatment (factors: ethanol cessation, variant/isoform frequency) or genotype (factors: genotype, variant/isoform frequency) on the frequency of transcript variants/protein isoforms. Following ANOVA, a post hoc Tukey test was applied. To control the type I error rate, comparisons were conducted using a significance level (*α*) of *p* < 0.05. Statistical analyses were performed using GraphPad Prism v.9.3.0 Software (GraphPad Software, La Jolla, CA, USA) or Statistica v.13.3 (TIBCO Software, Palo Alto, CA, USA).

## Results

### Ethanol intake in the C57BL/6N mouse strain

**Fig. 2:**
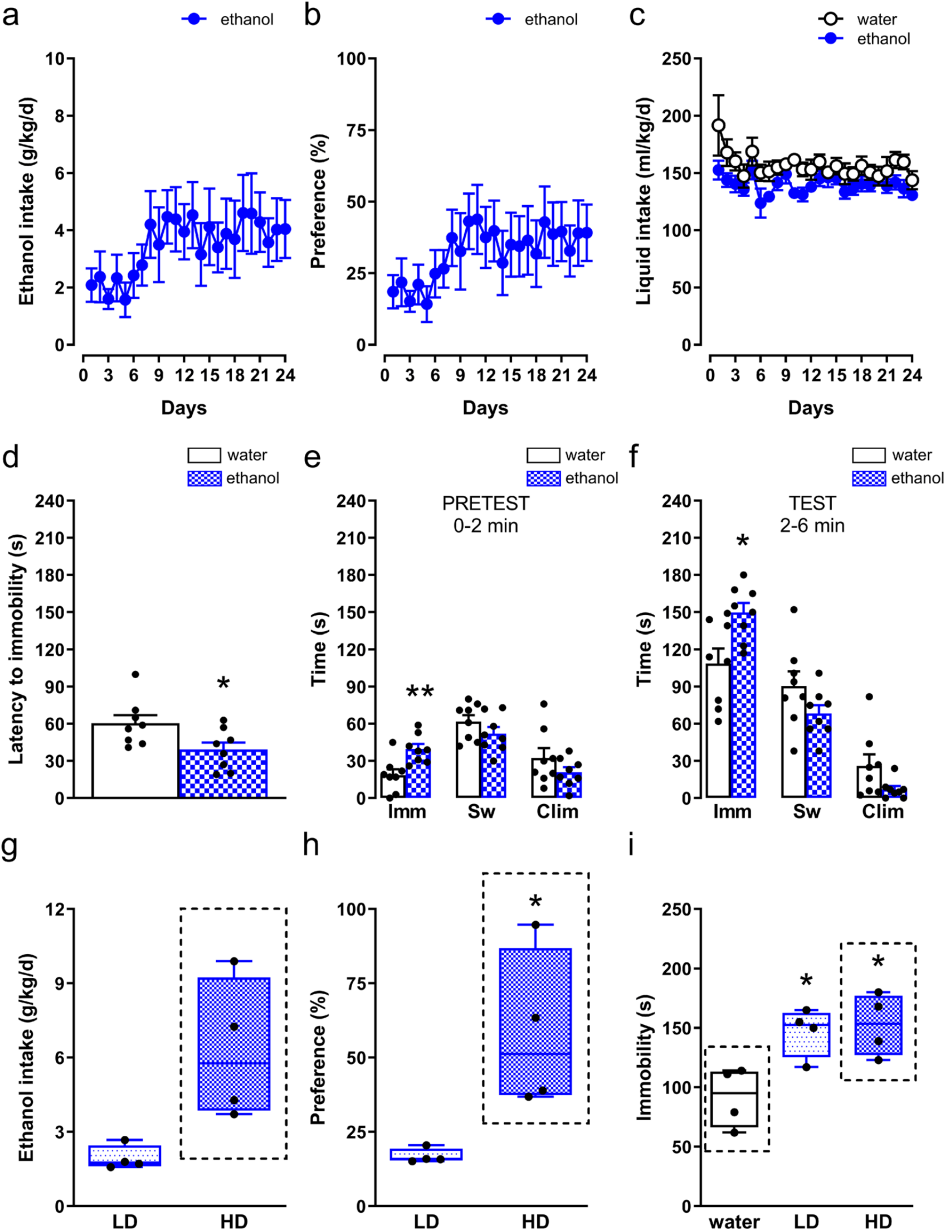
Behavioral analysis of C57BL/6N mice chronically consuming ethanol. The animals were given access to water and 10% ethanol (a two-bottle free-choice procedure) for 24 days (ethanol; **a** − **c**). Control mice had access to two water bottles (water). The bottles containing water and ethanol were weighed at 24-h intervals. Following 24 days, animals underwent a deprivation period. On day 3 of ethanol cessation, the phenotype of mice was assessed in the FST (**d** − **f**). Based on the amount of ethanol consumed within the final week of ethanol drinking, animals were divided into “high” (HD) and “low (LD) ethanol-drinking” groups. Ethanol-drinking parameters (**g**, **h**) and behavior in the FST (**i**) were compared between HD and LD groups. The control group consisted of animals drinking water. The measured parameters were as follows: **a** ethanol consumption − g of pure ethanol ingested within 1 day per kg of body weight, (g/kg/day); **b** preference for ethanol − g of ethanol consumed per day as the percentage of total liquid consumed, %; **c** liquid intake – ml of liquid (water or water + ethanol) ingested within 1 day per kg of body weight, (ml/kg/day); **d** latency to the first immobility, (s); duration of immobility (Imm, (s)), swimming (Sw, (s)), and climbing (Clim, (s)) during **e** the “PRETEST” and **f** “TEST”; **g** average ethanol consumption (last week of access to ethanol, (g/kg/day)); **h** average preference (last week of ethanol drinking, %); **i** immobility time during the “TEST” (in this particular comparison, the control group consisted of only those water-drinking animals that were selected for molecular analyses). **g** − **i** black dashed rectangles indicate groups of animals selected for molecular analyses. The data are presented as the means (± SEM) (*n* = 8 mice/group; **a** − **f**) or as box plots, with the horizontal line indicating the median, and vertical boxes and whiskers depicting the percentile range (*n* = 4 mice/group; **g** − **i**). **a**, **b** Repeated measures ANOVA followed by the Tukey test; **c** one-way ANOVA with repeated measures: *p* < 0.001: main effect for the day; **d** *t*-test: ^*^p < 0.05 vs. water; **e** *t*-test: ^**^p < 0.01 vs. water; **f** *t*-test: ^*^p < 0.05 vs. water; **g** *t*-test: ns; **h** Mann–Whitney *U* test: ^*^p < 0.05 vs. LD; **i** post hoc Tukey test: ^*^p < 0.05 vs. water

Ethanol intake in C57BL/6N mice was changed according to drinking days (*F*_23,161 _= 1.98, *p *= 0.0076, *η*^2^_*p *_= 0.22). However, as verified by the Tukey test, no changes in the level of ethanol consumption were found between all experimental days as compared to the initial ethanol exposure on day 1 (Fig. 2a). In total, mice consumed 83.52 ± 18.95 g/kg of pure ethanol during 24 days (average ethanol intake: 3.48 ± 0.79 g/kg/day).

Ethanol preference was changed depending on ethanol drinking days (*F*_23,161 _= 1.88, *p *= 0.013, *η*^2^_*p *_= 0.21). However, as verified by the Tukey test, no difference in the ethanol preference was found between all experimental days and the first day of ethanol exposure (Fig. 2b).

Ethanol drinking did not alter total intake of liquid (ethanol + water) throughout the drinking days (no ethanol treatment x day interaction (*F*_23,322 _= 0.90, *p *= 0.61, *η*^2^_*p *_= 0.060); Fig. 2c). There was a significant effect of the day (*F*_23,322 _= 3.17, *p *= 0.000003, *η*^2^_*p *_= 0.18), but no main effect of ethanol exposure (*F*_1,14 _= 4.22, *p *= 0.059, *η*^2^_*p *_= 0.23), on total liquid consumption (Fig. 2c).

### Mice deprived of ethanol exhibit enlarged immobility and decreased latency to the first immobility behavior

According to the analysis of animals’ behavior assessed in the FST, early (3 days) ethanol cessation in mice reduced latency to immobility compared to control mice exposed to water (*t*_14 _= 2.38, *p *= 0.032, *d *= 1.19; Fig. 2d).

A 3-day ethanol deprivation significantly increased (by 114%) immobility time in mice during the “pretest” (0–2 min) phase (*t*_14 _= 3.31, *p *= 0.0052, *d *= 1.66; Fig. 2e). Ethanol deprivation did not change swimming (*t*_14 _= 1.32, *p *= 0.21, *d *= 0.66) or climbing (*t*_14 _= 1.28, *p *= 0.22, *d *= 0.64) behaviors in animals during a 0–2-min measurement (Fig. 2e).

During the “test” (2–6 min), ethanol cessation significantly elevated (by 38%) immobility time (*t*_14 _= 2.84, *p *= 0.013, *d *= 1.42; Fig. 2f). In contrast, ethanol cessation did not alter swimming (*t*_14 _= 1.62, *p *= 0.13, *d *= 0.81) or climbing (*U *= 15.50, *p *= 0.088, *d *= 0.97) behaviors (Fig. 2f).

Overall, ethanol deprivation decreased latency and enhanced immobility time, suggesting the development of depression-like behavior during the early phase of ethanol cessation.

### High-ethanol drinkers display a higher preference for ethanol than low-ethanol drinkers

During the final week of 10% ethanol consumption, “high ethanol-drinking” mice displayed a trend (*t*_3.18 _= 3.00, *p *= 0.054, *d *= 2.12) towards an increased (by 3.2-fold) average ethanol intake compared to “low ethanol-drinking” animals (Fig. 2g). In this period, the high-ethanol drinkers displayed an enlarged (by 3.5-fold) preference for ethanol compared to low-ethanol drinkers (*U *= 0, *p *= 0.029, *d *= 2.17; Fig. 2h). Additional data regarding the pattern of ethanol drinking and ethanol preference during 24 days in high and low-ethanol drinkers are given in Online Resource 1.

Ethanol deprivation in mice divided into high and low-ethanol drinkers significantly changed the immobility time (*F*_2,9 _= 7.77, *p *= 0.011, *η*^2^_*p *_= 0.63; Fig. 2i). The post hoc Tukey test indicated that the immobility behavior in high and low-ethanol drinkers was significantly higher compared to water-drinking mice (*p *= 0.015 and *p *= 0.025, respectively; Fig. 2i). However, there was no difference in the level of immobility between the “high” and “low ethanol-drinking” groups (*p *= 0.94; Fig. 2i).

Taken together, the behavioral analysis of ethanol-drinking mice identified “high ethanol-drinking” animals that, compared to the “low ethanol-drinking” group, showed a higher preference for ethanol and a higher, but non-significant, ethanol consumption.

### “High ethanol-drinking” mice display reduced levels of *Tph2* mRNA in the hippocampus

To study the relationship between 5-HT synthesis and ethanol cessation, we evaluated the expression of *Tph2* in animals that had previously consumed high levels of ethanol and were subsequently subjected to ethanol deprivation.

**Fig. 3:**
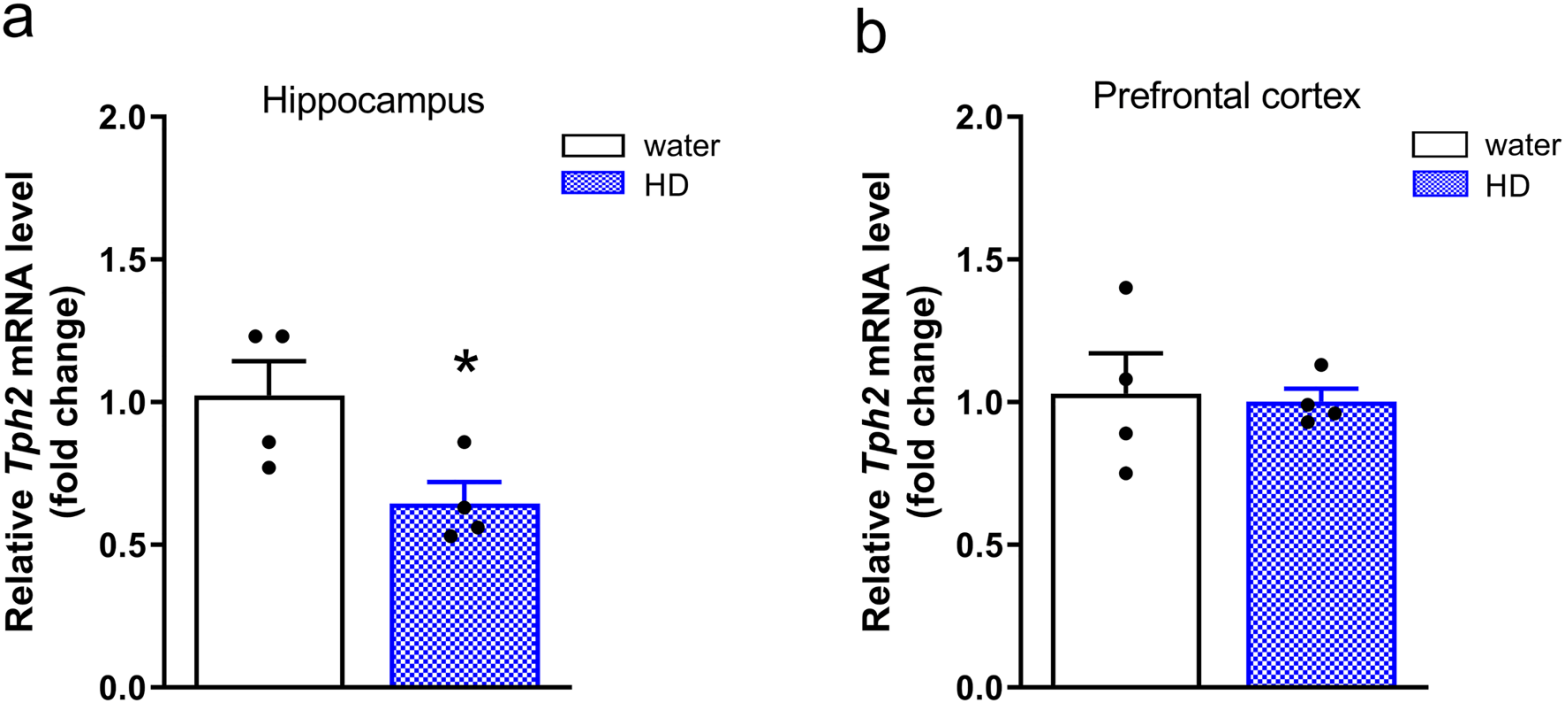
Analysis of *Tph2* expression in the hippocampi and prefrontal cortices of high-ethanol drinkers during early drug cessation. Animals were given access to water and 10% ethanol for 24 days. Control mice had access to two water bottles (water). After 24 days, the mice underwent a deprivation period. On day 3 of ethanol cessation, animals were subjected to the FST. After the test, the mice were sacrificed, and the brain tissue was isolated. Based on the amount of ethanol consumed, animals were divided into “high” (HD) and “low ethanol-drinking” groups. For molecular analyses, only the HD group (*n* = 4) and randomly selected water-drinking animals (*n* = 4) were included. The level of *Tph2* mRNA was determined in **a** the hippocampus and **b** the prefrontal cortex using qPCR. Data were normalized to *Tbp* and are presented as fold change (${2}^{-\Delta \Delta {\mathrm{C}}_{\mathrm{T}}}$) means (± SEM). **a** *t*-test: ^*^p < 0.05 vs. water; **b** *t*-test: ns

*Tph2* expression in the hippocampus of high-ethanol drinkers was reduced (by 37%) in comparison to the transcript level in the water-drinking group (*t*_6 _= 2.65, *p *= 0.038, *d *= 1.88; Fig. 3a). In the prefrontal cortex, there was no difference in the amount of *Tph2* mRNA between “high ethanol-drinking” and water-drinking mice (*t*_6 _= 0.19, *p *= 0.86, *d *= 0.13; Fig. 3b).

In general, ethanol cessation in mice consuming high levels of ethanol reduced the expression of *Tph2* in the hippocampus.

### Ethanol cessation elevates 5-HT_2C_ receptor mRNA editing in the hippocampus of “high ethanol-drinking” mice

**Fig. 4:**
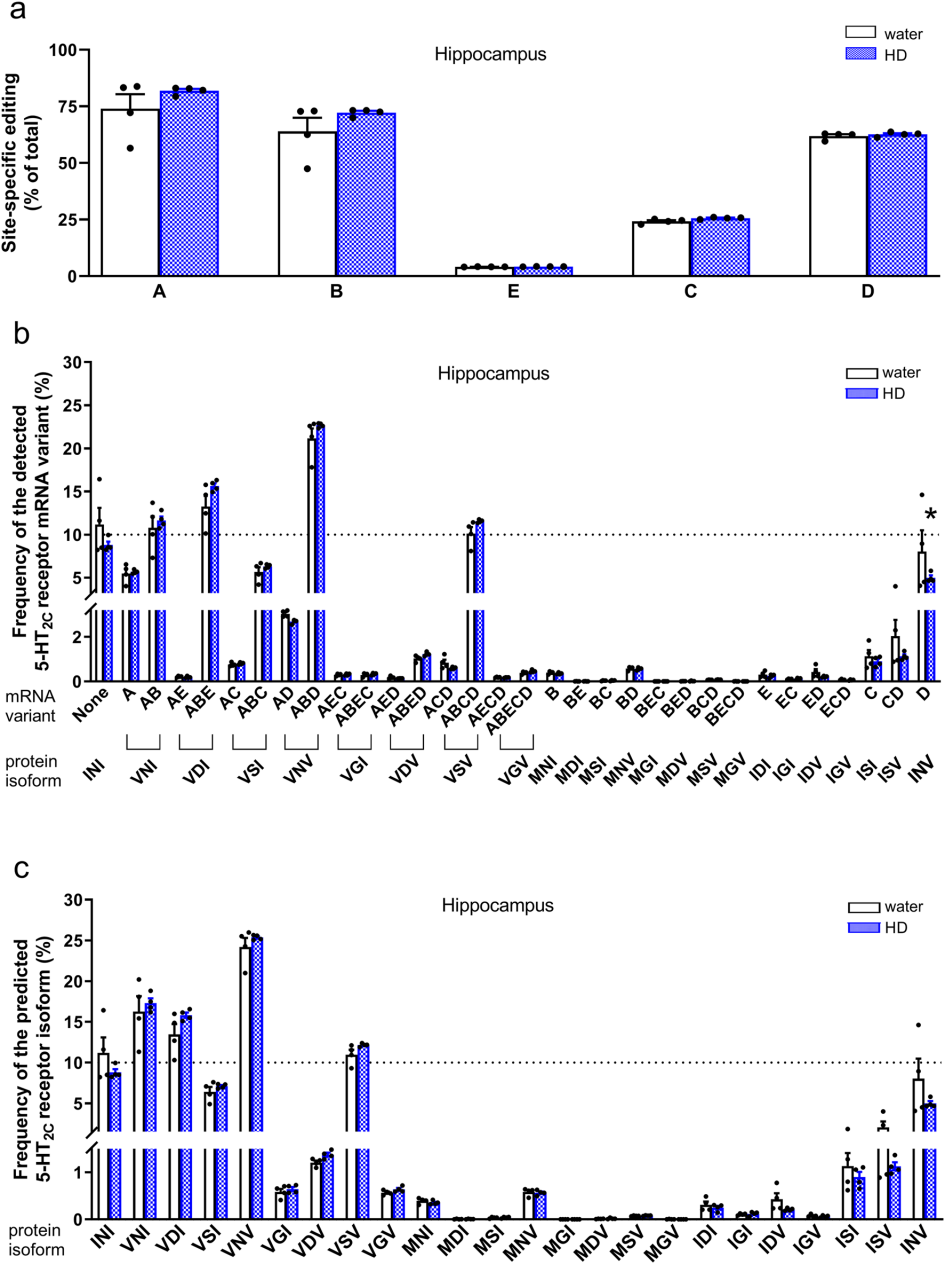
The 5-HT_2C_ receptor mRNA editing in the hippocampus of “high ethanol-drinking” mice during early drug cessation. Animals were given access to water and 10% ethanol for 24 days. Control animals had access to two water bottles (water). After 24 days, the mice underwent a period of ethanol deprivation. On day 3 of ethanol cessation, the phenotype of animals was evaluated in the FST. After the test, the mice were sacrificed, and the hippocampus was isolated. Based on the amount of ethanol consumed, animals were categorized as high (HD) and low ethanol drinkers. For molecular analyses, only the HD group (*n* = 4) and randomly selected water-drinking animals (*n* = 4) were included. The mRNA editing of the 5-HT_2C_ receptor was determined by deep sequencing. Data are presented as means (± SEM) for site-specific (labeled A to E) editing (**a**), frequency of the detected transcript variants (**b**), and predicted receptor isoforms (**c**). The mRNA variants resulting from editing at sites A − E (e.g., AC, ABD, ABCD) and the corresponding protein isoforms with amino acid substitutions at positions 156-158-160 (e.g., VSI, VNV, VSV) are indicated. **a** Two-way ANOVA: *p* < 0.05: main effect for the treatment (ethanol deprivation); *p* < 0.001: main effect for the editing site; **b** two-way ANOVA followed by the post hoc Tukey test: ^*^p < 0.05 vs. water; **c** two-way ANOVA: *p* < 0.001: main effect for the frequency of receptor isoforms

**Fig. 5:**
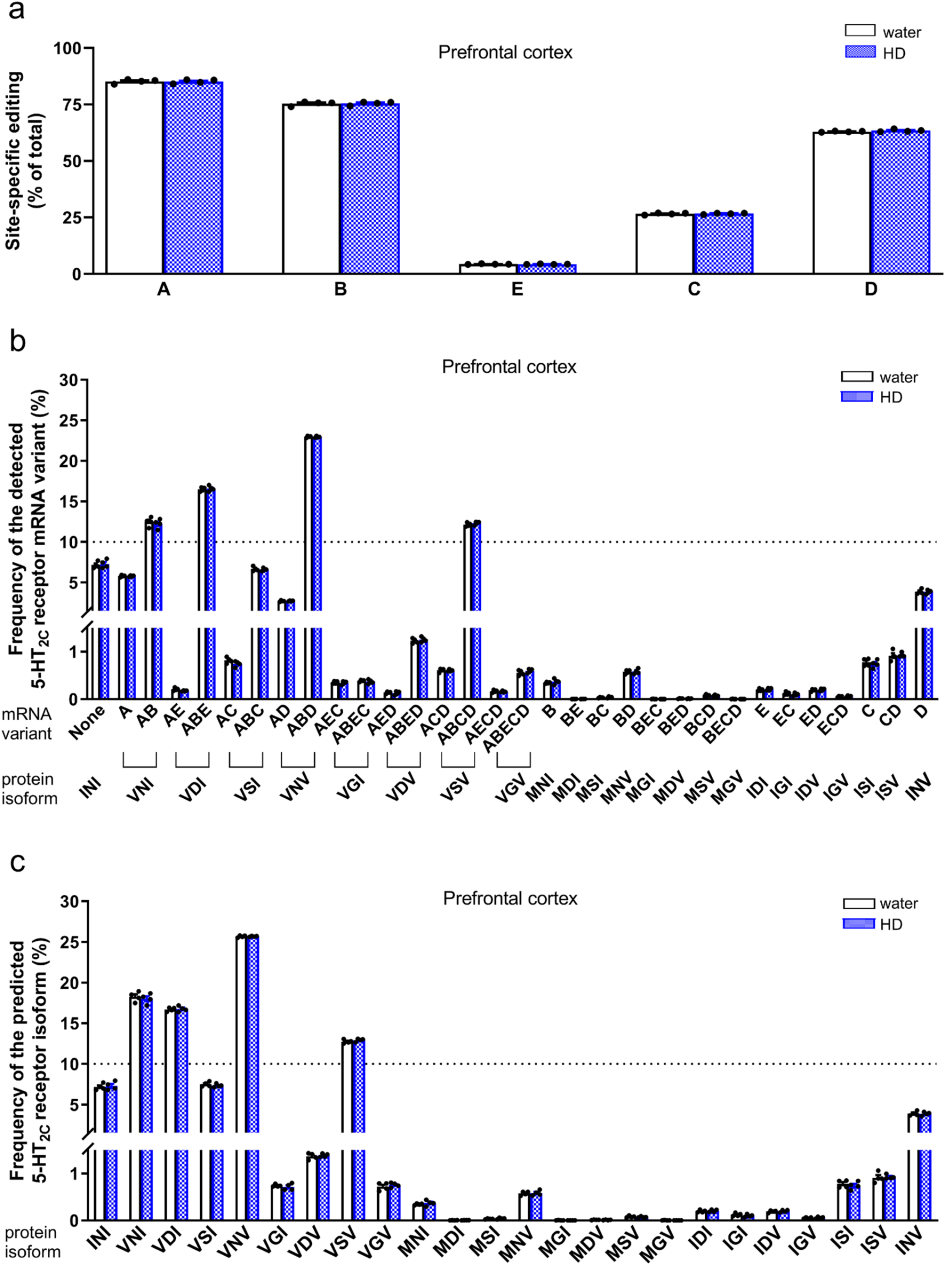
The mRNA editing of the 5-HT_2C_ receptor in the prefrontal cortex of “high ethanol-drinking” mice during early drug cessation. Animals were given access to water and 10% ethanol for 24 days. Control animals had access to two water bottles (water). After 24 days, the mice underwent a period of ethanol deprivation. On day 3 of ethanol cessation, the phenotype of mice was evaluated in the FST. After the test, the mice were sacrificed, and the prefrontal cortex was isolated. Based on the amount of ethanol consumed, animals were categorized as high (HD) and low ethanol drinkers. For molecular analyses, only the HD group (*n* = 4) and randomly selected water-drinking animals (*n* = 4) were included. The mRNA editing of the 5-HT_2C_ receptor was determined by deep sequencing method. Data are presented as means (± SEM) for site-specific (labeled A to E) editing (**a**), frequency of the detected transcript variants (**b**), and predicted receptor isoforms (**c**). The mRNA variants resulting from editing at sites A − E (e.g., AC, ABD, ABCD) and the corresponding protein isoforms with amino acid substitutions at positions 156–158-160 (e.g., VSI, VNV, VSV) are indicated. **a** Two-way ANOVA: *p* < 0.001: main effect for the editing site; **b** two-way ANOVA: *p* < 0.001: main effect for the frequency of mRNA variants; **c** two-way ANOVA: *p* < 0.001: main effect for the frequency of receptor isoforms

Deep sequencing of the mRNA editing of the 5-HT_2C_ receptor in the prefrontal cortex and hippocampus of control mice revealed that the most intense modification was reported at sites A, B and D (>60%), modest at site C (ca. 25%) and the lowest at site E (ca. 4%) (Figs. 4a, 5a). Statistical analysis showed that the effect of brain region on site-specific editing did not change according to editing site (no brain region x editing site interaction (*F*_4,30 _= 2.01, *p *= 0.12, *η*^2^_*p *_= 0.21)). However, a significant main effect for the brain region (*F*_1,30 _= 8.92, *p *= 0.0056, *η*^2^_*p *_= 0.23) and editing site (*F*_4,30 _= 262.30, *p *< 0.001, *η*^2^_*p *_= 0.97) on site-specific editing were reported (Figs. 4a, 5a). These results indicated that, regardless of the editing site, the global 5-HT_2C_ receptor editing level in the prefrontal cortex of water-drinking mice was higher than in the hippocampus.

In the hippocampus of control animals, the ABD, ABE, None, AB, and ABCD were the most frequent (ca. 10−21%) transcript variants (Fig. 4b). In the case of the predicted receptor isoforms, the VNV, VNI, VDI, INI, and VSV displayed the highest frequency (ca. 11–24%) (Fig. 4c).

The analysis revealed that ethanol cessation in mice previously consuming high levels of ethanol did not change site-specific editing for the mRNA of 5-HT_2C_ receptor in the hippocampus according to editing site (the absence of an effect of treatment x editing site interaction (*F*_4,30 _= 1.03, *p *= 0.41, *η*^2^_*p *_= 0.12)). However, significant main effects were observed for the treatment (*F*_1,30 _= 4.28, *p *= 0.047, *η*^2^_*p *_= 0.12) and editing site (*F*_4,30 _= 249.15, *p *< 0.001, *η*^2^_*p *_= 0.97) (Fig. 4a). This analysis demonstrated that ethanol deprivation, regardless of the editing site, increased the level of site-specific editing in the hippocampus.

The analysis of the occurrence of 5-HT_2C_ receptor transcript variants demonstrated that a 3-day ethanol deprivation in high-ethanol drinkers significantly affected the frequency of the detected mRNA variants in the hippocampus (a significant treatment x mRNA variant frequency interaction (*F*_31,192 _= 1.67, *p *= 0.02, *η*^2^_*p *_= 0.21); Fig. 4b). The post hoc Tukey test showed that ethanol cessation reduced the frequency of the D mRNA variant compared to the water-drinking group (*p *= 0.037; Fig. 4b), suggesting an increase in the corresponding INV receptor isoform. However, the analysis of predicted receptor protein isoforms in the hippocampus did not show any alterations in the frequency of receptor isoforms in ethanol-deprived animals (a significant effect of receptor isoform frequency (*F*_23,144 _= 237.47, *p *< 0.001, *η*^2^_*p *_= 0.97), but no main effect of the treatment (*F*_1,144 _= 0, *p *= 1, *η*^2^_*p *_= 0) or treatment x receptor isoform frequency interaction (*F*_23,144 _= 1.48, *p *= 0.087, *η*^2^_*p *_= 0.19); Fig. 4c).

In the prefrontal cortex of control animals, the ABD, ABE, AB, and ABCD were the most frequent (ca. 12−23%) mRNA variants (Fig. 5b). In the case of the predicted receptor isoforms, the VNV, VNI, VDI, and VSV exhibited the highest frequency (ca. 13–26%) (Fig. 5c).

According to the two-way ANOVA analysis, a short-term ethanol cessation did not have an effect on site-specific editing in the prefrontal cortex of high-ethanol drinkers (a significant effect of editing site (*F*_4,30 _= 26189.7, *p *< 0.001, *η*^2^_*p *_= 0.9997), but no effect of treatment (*F*_1,30 _= 0.3, *p *= 0.61, *η*^2^_*p *_= 0.0087) or treatment x editing site interaction (*F*_4,30 _= 0.2, *p *= 0.92, *η*^2^_*p *_= 0.029); Fig. 5a).

Analyses of the frequency of receptor mRNA variants in the prefrontal cortex revealed that ethanol deprivation did not induce changes in the frequency of mRNA variants (a significant effect of mRNA variant frequency (*F*_31,192 _= 9354.23, *p *< 0.001, *η*^2^_*p *_= 0.999), but no effect of treatment (*F*_1,192 _= 0, *p *= 1, *η*^2^_*p *_= 0) or treatment x mRNA variant frequency interaction (*F*_31,192 _= 0.32, *p *= 0.9998, *η*^2^_*p *_= 0.05); Fig. 5b). Furthermore, there was no effect of ethanol deprivation on the frequency of predicted receptor isoforms in the prefrontal cortex (a significant effect of receptor isoform frequency (*F*_23,144 _= 10864.53, *p *< 0.001, *η*^2^_*p *_= 0.999), but no effect of treatment (*F*_1,144 _= 0, *p *= 1, *η*^2^_*p *_= 0) or treatment x receptor isoform frequency interaction (*F*_23,144 _= 0.34, *p *= 0.998, *η*^2^_*p *_= 0.051); Fig. 5c).

Altogether, after a short-term cessation from high ethanol drinking, mice displayed a global increase in the efficiency of site-specific editing in the hippocampus, which was accompanied by a reduction in the less common D mRNA variant.

### *Tph2* deficiency evokes distinct alterations in the mRNA editing pattern of the 5-HT_2C_ receptor in the hippocampus and prefrontal cortex

**Fig. 6:**
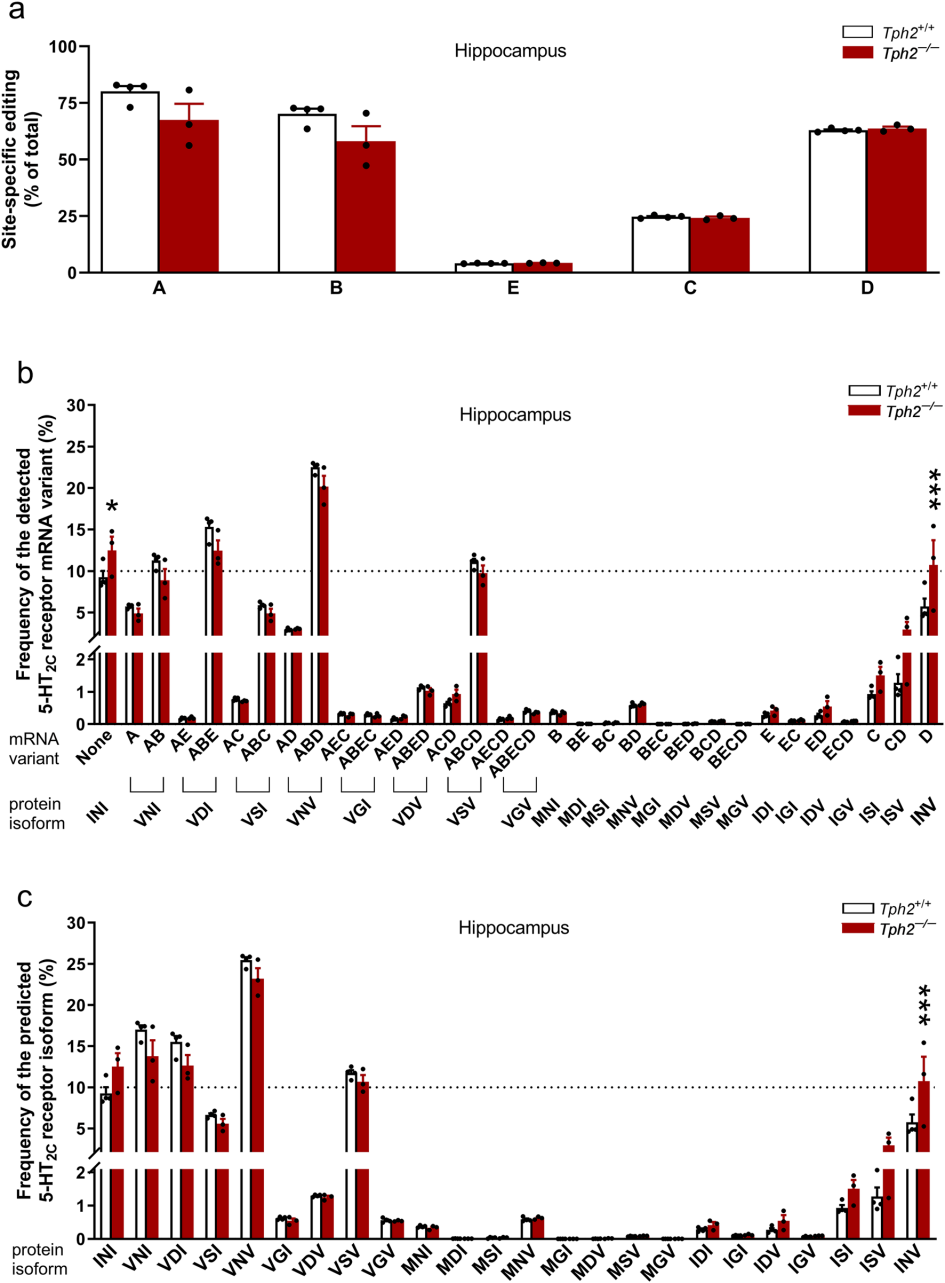
The 5-HT_2C_ receptor mRNA editing in the hippocampus of *Tph2*^*−/−*^ mice. Individually housed *Tph2*^*−/−*^ and *Tph*2^+/+ ^mice were given unrestricted access to two water bottles for 29 days. After ca. four weeks, the behavior of mice was evaluated in the FST. After the test, the mice were sacrificed, and the hippocampi were isolated. The mRNA editing of the 5-HT_2C_ receptor was evaluated by deep sequencing method. Data are shown as means (± SEM) for site-specific (labeled A to E) editing (**a**), frequency of the detected transcript variants (**b**), and predicted receptor isoforms (**c**) of data from 3 to 4 mice/group. The mRNA variants resulting from editing at sites A − E (e.g., AC, ABD, ABCD) and the corresponding protein isoforms with amino acid substitutions at positions 156-158-160 (e.g., VSI, VNV, VSV) are indicated. **a** Two-way ANOVA: p < 0.05: main effect for the genotype; *p* < 0.001: main effect for the editing site; **b** two-way ANOVA followed by the post hoc Tukey test: ^*^p < 0.05, ^***^p < 0.001 vs. *Tph2*^+*/*+^; **c** two-way ANOVA followed by the post hoc Tukey test: ^***^p < 0.001 vs.* Tph2*^+*/*+^

**Fig. 7:**
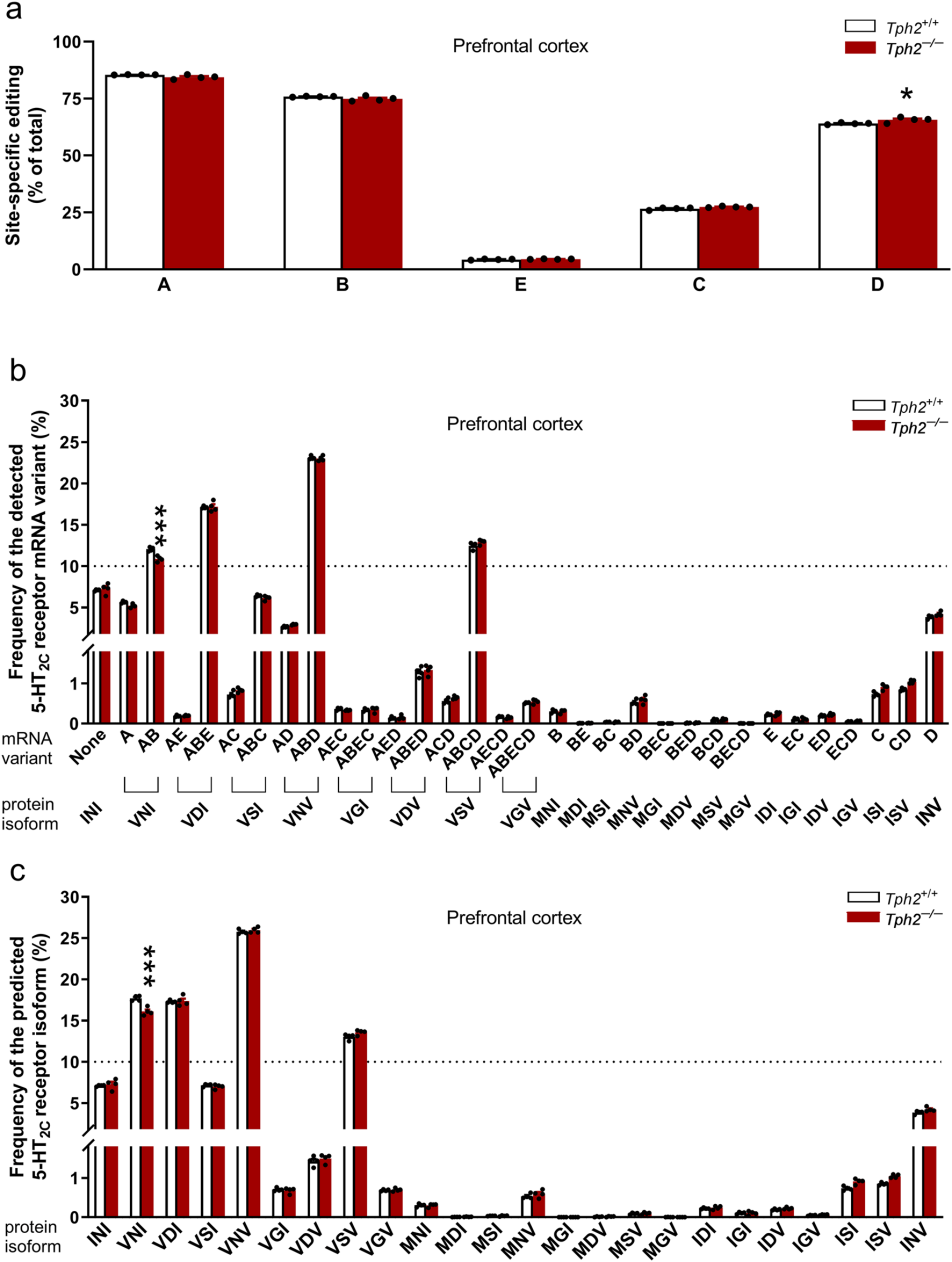
The editing of 5-HT_2C_ receptor mRNA in the prefrontal cortex of *Tph2*^*−/−*^ mice. Individually housed *Tph2*^*−/−*^ and *Tph2*^+/+^ mice remained in their home cages with unrestricted access to two water bottles for 29 days. After ca. four weeks, the behavior of mice was evaluated in the FST. After the test, the animals were sacrificed, and the prefrontal cortex was isolated. The mRNA editing of the 5-HT_2C_ receptor was determined by deep sequencing. Data are presented as means (± SEM) for site-specific (labeled A to E) editing (**a**), frequency of the detected transcript variants (**b**), and the predicted receptor isoforms (**c**) of data from 4 mice/group. The mRNA variants resulting from editing at sites A − E (e.g., AC, ABD, ABCD) and the corresponding protein isoforms with amino acid substitutions at positions 156-158-160 (e.g., VSI, VNV, VSV) are indicated. **a** Two-way ANOVA followed by the post hoc Tukey test: ^*^p < 0.05 vs.* Tph2*^+*/*+^; **b** two-way ANOVA followed by the post hoc Tukey test: ^***^p < 0.001 vs. *Tph2*^+*/*+^; **c** two-way ANOVA followed by the post hoc Tukey test: ^***^p < 0.001 vs. *Tph2*^+*/*+^

In the prefrontal cortex and hippocampus of *Tph2*^+/+^ mice, the most intense editing was observed at sites A, B, and D (>60%), modest at site C (ca. 26%), and the lowest at site E (ca. 4%) (Figs. 6a, 7a). In these control mice, the effect of brain region on site-specific editing for the mRNA of 5-HT_2C_ receptor significantly changed according to the editing site (a significant effect of brain region x editing site interaction (*F*_4,30 _= 2.88, *p *= 0.039, *η*^2^_*p *_= 0.28), brain region (*F*_1,30 _= 18.69, *p *= 0.00016, *η*^2^_*p *_= 0.38), and editing site (*F*_4,30 _= 2067.59, *p *< 0.0001, *η*^2^_*p *_= 0.996); Figs. 6a, 7a). The Tukey test indicated that the editing at sites A and B in the prefrontal cortex was higher than that in the hippocampus (A: *p *= 0.029; B: *p *= 0.018; Figs. 6a, 7a). No significant difference in the editing level at sites E, C or D was found between the two tested brain regions (*p *> 0.05; Figs. 6a, 7a).

In both tested brain regions of *Tph2*^+*/*+^ mice, the ABD, ABE, AB, and ABCD were the most frequent (ca. 11−23%) transcript variants (Figs. 6b, 7b). Correspondingly, the VNV, VNI, VDI, and VSV protein isoforms displayed the highest frequency (ca. 12–26%) (Figs. 6c, 7c).

Although the two-way ANOVA analysis suggested that *Tph2* deficiency influenced site-specific editing for the mRNA of 5-HT_2C_ receptor in the hippocampus based on the editing site (a significant genotype x editing site interaction (*F*_4,25 _= 2.87, *p *= 0.044, *η*^2^_*p *_= 0.31)), the Tukey test indicated no differences (*p *> 0.05) in editing at any of the five editing sites between *Tph2*^–/–^ and *Tph2*^+/+^ mice (Fig. 6a). However, the analysis revealed a significant main effect for genotype (*F*_1,25 _= 7.13, *p *= 0.013, *η*^2^_*p *_= 0.22) and editing site (*F*_4,25 _= 217.13, *p *< 0.0001, *η*^2^_*p*_ = 0.97), indicating that *Tph2* deficiency reduced the overall efficiency of site-specific editing in the hippocampus, regardless of the specific editing site.

The analysis of the abundance of hippocampal 5-HT_2C_ receptor transcript variants showed that *Tph2* deficiency significantly changed the frequency of mRNA variants (a significant genotype x mRNA variant frequency interaction (*F*_31,160 _= 3.77, *p *< 0.0001, *η*^2^_*p *_= 0.42); Fig. 6b). The post hoc Tukey test demonstrated that *Tph2*^–/–^ mice displayed an increased frequency of the non-edited (None; *p *= 0.013) and D (*p *= 0.000027) mRNA variants, and a trend towards a decrease (*p *= 0.089) in the abundance of the ABE mRNA variant compared to *Tph2*^+/+^ mice (Fig. 6b).

Further analysis showed that *Tph2* deficiency affected the frequency of the predicted 5-HT_2C_ receptor isoforms in the hippocampus (a significant genotype x receptor isoform frequency interaction (*F*_23,120 _= 3.74, *p *= 0.000001, *η*^2^_*p *_= 0.42); Fig. 6c). The post hoc Tukey test revealed that *Tph2* deficiency increased the frequency of the predicted INV receptor isoform (*p *= 0.00021; Fig. 6c).

The analysis revealed that the impact of depletion of central 5-HT on 5-HT_2C_ receptor mRNA site-specific editing in the prefrontal cortex significantly varied across different editing sites (a significant genotype x editing site interaction (*F*_4,30 _= 6.8, *p *= 0.00051, *η*^2^_*p *_= 0.48); Fig. 7a). The post hoc Tukey test showed that *Tph2* deficiency led to an elevation in editing at site D (*p *= 0.028), but not at the other four editing sites (i.e., A, B, E or C; *p* > 0.05) in the prefrontal cortex (Fig. 7a). At the same time, depletion of central 5-HT in *Tph2*^*−/−*^ mice affected the frequency of mRNA variants in the prefrontal cortex (a significant genotype x mRNA variant frequency interaction (*F*_31,192 _= 5.11, *p *< 0.0001, *η*^2^_*p *_= 0.45); Fig. 7b). The post hoc Tukey test showed a reduction in the frequency of the AB mRNA variant in *Tph2*^*−/−*^ mice compared to *Tph2*^+*/*+^ mice (*p *= 0.000027; Fig. 7b). Further analysis showed that 5-HT depletion in *Tph2*^*−/−*^ animals significantly altered the frequency of predicted receptor isoforms in the prefrontal cortex (a significant genotype x receptor isoform frequency interaction (*F*_23,144 _= 7.15, *p *< 0.0001, *η*^2^_*p *_= 0.53); Fig. 7c). The post hoc Tukey test demonstrated that *Tph2* deficiency led to a decrease in the VNI receptor isoform frequency (*p *= 0.00005) and a trend towards an increase in the abundance of the VSV receptor isoform (*p *= 0.08) (Fig. 7c).

Overall, *Tph2* deficiency induced distinct changes in editing of 5-HT_2C_ receptor in the brain: a global decrease in editing efficiency in the hippocampus accompanied by increased levels of more active receptor isoforms, and in the prefrontal cortex, an increase in editing at site D coupled with a reduced frequency of the VNI receptor isoform.

## Discussion

Our study demonstrated that abrupt cessation of high ethanol consumption in mice induced global enhancement of the 5-HT_2C_ receptor mRNA editing in the hippocampus but not the prefrontal cortex. The changes in receptor editing during ethanol cessation can be explained by dysregulation of central 5-HT synthesis, as measured by reduced levels of *Tph2* expression in the hippocampus. We also showed that the 5-HT_2C_ receptor mRNA editing in the hippocampus and prefrontal cortex is sensitive to the life-long inhibition of 5-HT synthesis in the brain.

In this research, we applied a two-bottle drinking procedure with everyday access to 10% ethanol for 24 days in mice. This model has been extensively utilized in the literature to investigate the behavioral and biochemical correlates of voluntary ethanol intake [53–55, 58]. Our research focused on C57BL/6NCrl mice, which exhibited moderate levels of ethanol intake (ca. 3.5 g/kg/day) compared to previous studies using the same ethanol concentration and mouse substrain (> 10 g/kg/day; [59]). It must be noted that C57BL/6NCrl mice are considered to have low preference for ethanol when compared to the high ethanol-preferring C57BL/6 J mouse substrain [60]. However, these mice provide a suitable model for studying ethanol craving as short-term ethanol deprivation in this strain can induce a deprivation effect characterized by elevated ethanol consumption and preference after re-exposure to ethanol [61]. Our findings revealed that upon the discontinuation of ethanol intake, specifically during the early phase of drug cessation (day 3), mice exhibited enhanced immobility behavior and decreased latency to the first immobility in the FST. Notably, no significant alterations in swimming and climbing behaviors were reported in ethanol-deprived mice. These results indicate the development of a depression-like phenotype in mice undergoing ethanol abstinence, which aligns with previous studies showing a depression-like response characterized by enhanced immobility on day 1 and/or 14 of ethanol cessation in other mouse strains, including C57BL/6 J mice [15, 54, 62].

In the present investigation, we employed a previously established method of categorizing animals based on their individual differences in ethanol consumption during the last week of access to ethanol [5]. By employing this method, we were able to identify two distinct groups of animals: “high ethanol-drinking” mice consuming ca. 6 g/kg/day of ethanol, and “low ethanol-drinking” mice, consuming ca. 2 g/kg/day of ethanol. Strikingly, despite their distinct levels of ethanol intake and preference, both high and low-ethanol drinkers showed a similar degree of depression-like response in the FST during drug cessation. This finding suggests that the development of depressed mood in ethanol-deprived mice does not depend on the amount of ethanol consumed before deprivation. It is important to note that the FST primarily assesses one depression symptom, ‘entrapment’ [63]. To establish the similarity in the severity of depression between high and low-ethanol drinkers, additional research is required to determine whether other depression symptoms, such as anhedonia or sleep disturbances, can be observed in these drug-weaned animals.

To unravel the mechanisms underlying ethanol dependence and to identify molecular factors potentially linked to relapse, we opted to conduct molecular analyses exclusively in “high ethanol-drinking” mice. These mice exhibited a pattern of increased ethanol consumption over the course of 24 days. Conversely, we chose to exclude mice that did not display changes in ethanol intake and preference (i.e., “low ethanol-drinking” mice) over 24 days of ethanol exposure.

Importantly, this study provides the first evidence of significant changes in 5-HT_2C_ receptor mRNA editing within the hippocampus, rather than the prefrontal cortex, among ethanol-deprived “high ethanol-drinking” animals. These observations are in line with an earlier study demonstrating alterations in the density of 5-HT_2C_ receptors in the hippocampus of alcohol-preferring rats in comparison to rats with low alcohol preference, while no changes were found in the cortex [64]. Thus, our study supports the notion that long-term exposure to ethanol and/or its deprivation exerts a modulatory effect on hippocampal 5-HT_2C_ receptors.

Consistent with previous studies conducted on various mouse strains, including the C57BL/6 J substrain [30, 38, 65], we found that in the hippocampi and prefrontal cortices of control C57BL/6NCrl mice, the A, B, and D sites displayed the highest levels of editing (> 60%), and the VNV isoform was the predominant 5-HT_2C_ receptor isoform. However, noteworthy differences were observed in the basal efficiencies of site-specific editing and the frequencies of mRNA variants/predicted receptor isoforms in our C57BL/6NCrl strain when compared to other mouse strains exhibiting varying preferences for ethanol [30, 37, 38, 65]. For instance, the frequency of the highly functioning non-edited INI receptor isoform in the hippocampus or prefrontal cortex of C57BL/6NCrl mice (9% and 7%, respectively) resembled those observed in the amygdala (9.9%), nucleus accumbens (8%) or dorsal raphe nucleus (7%) of ethanol-avoiding DBA/2 J mouse strain [30, 38]. However, it was significantly higher than the levels of INI in the amygdala (1.35%), neocortex (2%), or nucleus accumbens (3%), but similar to the level in the dorsal raphe nucleus (7%) of high ethanol-preferring C57BL/6 J mice [30, 37, 38, 65]. Interestingly, previous research has indicated that exclusive expression of the INI isoform in C57BL/6 J mice can decrease enhanced ethanol drinking behavior in this ethanol-preferring mouse strain [37, 38]. Collectively, these observations suggest that the level of INI may regulate ethanol drinking behavior, but, even a higher level (9%) of INI does not prevent animals from developing increased ethanol consumption—as found in C57BL/6NCrl mice—implying the involvement of other receptors isoforms in controlling ethanol drinking behavior.

In the hippocampus of “high ethanol-drinking” mice undergoing drug deprivation, we reported an elevation in site-specific editing and a decrease in the frequency of the D transcript variant coding for the INV receptor isoform. However, the frequency analysis of all 24 predicted protein isoforms of the 5-HT_2C_ receptor showed no changes in the abundance of the INV receptor isoform. Although one could speculate that this change in the frequency of the D transcript may not be significant at the protein level, further research is required to verify the expression of all receptor protein isoforms.

In C57BL/6 J mice, voluntary ethanol intake has been found to increase the frequency of the low-activity receptor isoform, VNV, in the nucleus accumbens and dorsal raphe nucleus, but not in the hippocampus [37, 38], suggesting that ethanol-drinking behavior is associated with an elevation of less functional receptor isoforms in these brain regions. Currently, there is a lack of literature data on the editing status of accumbal and dorsal raphe 5-HT_2C_ receptors during ethanol abstinence, highlighting the need for further research in this area. However, based on our observation that ethanol deprivation, rather than chronic exposure to ethanol [38], enhanced the efficiency of the post-transcriptional editing process in the hippocampus, we may assume that the 5-HT_2C_ receptor editing profile in the hippocampus could serve as a hallmark of ethanol cessation in high ethanol-preferring animals.

In the context of neurotransmitter abnormalities associated with the cessation of high ethanol consumption and alterations in 5-HT_2C_ receptor mRNA editing, our present study focused on examining the 5-HT system in the brain. As previously demonstrated in a paradigm similar to our study, on day 14 of ethanol cessation, reductions in the tissue content of 5-HT were observed in the hippocampus but not the prefrontal cortex of C57BL/6 J mice [15]. The drug that reversed the elevated immobility time during early (day 1) and long-term (day 14) ethanol abstinence was also shown to attenuate the reduction in 5-HT levels in the hippocampus during the long-term drug cessation, indicating the modulatory role of hippocampal 5-HT neurotransmission in depression-like behavior during the persistent ethanol deprivation. Although we did not measure hippocampal 5-HT levels directly during early ethanol cessation in the current study, the observed reduction (by 37%) in *Tph2* expression in this brain region on the third day of ethanol abstinence suggests that short-term ethanol deprivation, associated with changes in 5-HT_2C_ receptor editing in the hippocampus, may be accompanied by changes in the local 5-HT synthesis in this brain area. In the prefrontal cortex, where no alteration in *Tph2* transcript level was observed during early ethanol cessation, no remodeling in the editing process was reported. Therefore, these observations indicate that alterations in 5-HT_2C_ receptor editing in the hippocampus observed during the early phase of ethanol deprivation are under the regulatory control of *Tph2* expression.

Previously, we have shown that mice consuming high amounts of ethanol showed higher levels of *Tph2* mRNA in the raphe nuclei and attenuated *Tph2* expression in the prefrontal cortex in comparison to low ethanol-drinking and/or water-drinking animals; the level of *Tph2* transcript in the hippocampus did not change after chronic ethanol intake [5]. Thus, we may assume that changes in the *Tph2* mRNA level detected in the present study in the hippocampus appear to be specifically related to the processes occurring during early ethanol cessation. Interestingly, mice with varying ethanol preferences (DBA/2 J, BALB/cJ and C57BL/6 J) exhibit functional SNP variations in the *Tph2* gene, resulting in differences in brain 5-HT content and synthesis [66]. To confirm whether the observed in our study reduction in *Tph2* transcript levels in the hippocampus following ethanol cessation corresponds to a decrease in 5-HT content, as evidenced in earlier studies [67, 68], it is necessary to assess hippocampal 5-HT levels through in vivo microdialysis. Certainly, other neurotransmitter systems, such as glutamate could be also related to ethanol deprivation-induced remodeling of the 5-HT_2C_ receptor RNA editing in the hippocampus [69]. Further investigation will be required to better understand the hippocampal microenvironment in ethanol-deprived mice.

In the present study, we show for the first time that complete depletion of 5-HT in the brain of *Tph2*^*−/−*^ mice induced significant alterations in 5-HT_2C_ receptor editing in the hippocampus and prefrontal cortex, suggesting that the 5-HT_2C_ receptor undergoes editing in the absence of its endogenous ligand, 5-HT. A global reduction in site-specific receptor editing in the hippocampus of *Tph2*^*−/−*^ mice was accompanied by an increase in the highly functional non-edited None transcript and less edited D mRNA variant encoding the INV receptor isoform. In the prefrontal cortex, *Tph2* deficiency led to an increased 5-HT_2C_ receptor RNA editing at site D and a reduction in the proportion of AB-edited mRNA variant corresponding to the predicted decrease in the frequency of the VNI protein isoform.

*Tph2*^*−/−*^ mice exhibited typical characteristics of central 5-HT depletion, such as growth restriction, heightened aggressive behavior, maternal neglect, and impaired social communication [48, 49, 70]. Despite evaluation by many research groups, a clear depression-like phenotype was not confirmed in *Tph2*^*−/−*^ mice [5, 49, 71–73]. In addition, naive *Tph2*^*−/−*^ mice showed increased water and food consumption [5, 74], while exposure to ethanol normalized water intake levels and increased ethanol consumption in these knockout animals [5]. *Tph2* deficiency did not block the antidepressant effect induced by chronic ethanol consumption [5]. As previously suggested [5], *Tph2*^*−/−*^ mice hold promise as a new model for ethanol dependence and exploring non-serotonergic drugs for the treatment of alcoholism.

So far, the data on the role of INV and VNI receptor isoforms, which were changed in the hippocampus and prefrontal cortex of *Tph2*^*−/−*^ mice, primarily stems from in vitro studies. It was found that a change in amino acid 161 in the INV isoform slightly lowered basal receptor activity compared to the non-edited INI isoform [33]. Editing at amino acid 157 in the VNI isoform led to a loss of functional selectivity of receptor agonists for the phospholipase A2 signaling pathway and a reduction in ligand-independent receptor activity [75]. However, another group did not observe any changes in receptor activity associated with editing in the VNI isoform [38]. These discrepancies could be potentially due to different phenotypes of the cell lines used by the research groups. Based on the above-described in vitro studies, it may be assumed that 5-HT deficiency in the mouse brain could reorganize the 5-HT_2C_ receptor editing pattern by modifying the frequency of receptor isoforms with lower constitutive activity. To validate the current findings on the frequency of 5-HT_2C_ receptor variants in the brain of *Tph2*^*−/−*^ mice, it would be essential to evaluate the basal and agonist-stimulated responsiveness of 5-HT_2C_ receptors in these knockout animals.

Literature data regarding the effects of dysregulated 5-HT neurotransmission on 5-HT_2C_ receptor editing are inconsistent. In contrast to our observations, another study found no significant influence of 5-HT depletion in the brain on the 5-HT_2C_ receptor RNA editing in the hippocampus of *Pet-1* knockout mice devoid of neurons producing 5-HT [76]. Such discrepancy between the studies might be due to the extent of the depletion of 5-HT in the brains of these knockout animals. Disruption of *Pet-1* produced a loss in 5-HT neurons and neurotransmitter levels (85–90%) in the brain [77], while inactivation of the *Tph2* gene in mice used in the present study led to almost complete (> 98%) lowering in 5-HT in the brain [48]. Other researchers assessing the editing changes in the forebrain neocortex (brain region including the prefrontal cortex) have shown that pharmacological 5-HT depletion by *para*-chlorophenylalanine (*p*CPA), an irreversible inhibitor of both TPH (1 and 2) isoenzymes decreased RNA editing at sites C and E, increased the frequency of ABD (encoding the VNV isoform) and decreased transcripts that include editing at sites E and EC [29]. The inconsistencies between the studies are likely to be related to the specificity of tools that were used to induce 5-HT depletion (genetic deletion of the gene coding for TPH2; the present study vs. inhibition of TPH1/2 by *p*CPA; [29]), duration of 5-HT depletion (a life-long; the present study vs. short-term; [29]) or the scope of the brain region analyzed (the prefrontal cortex; the present study vs. forebrain neocortex; [29]). In turn, other studies have demonstrated that a three to fourfold increase in extracellular 5-HT levels in mice deficient in serotonin transporter (SERT^*−/−*^) resulted in an elevated frequency of more edited (ABD, ABCD) and decreased abundance of less edited (None, BD, and D) mRNA variants in the amygdala [31], but did not induce changes in receptor RNA editing in the ventral hippocampus or medial prefrontal cortex [78]. The latter findings correspond well with our observations, suggesting that complete depletion of 5-HT in the brain attenuated the 5-HT_2C_ receptor editing process and shifted the receptor profile into the less edited mRNA variants (an increase in None and D, and a decrease in AB) showing a higher functioning profile. Conversely, the elevation in 5-HT neurotransmission enhanced the RNA editing process, leading to an increased frequency of less functioning receptor variants (an increase in ABD and ABCD, and a decrease in None, and D).

In conclusion, our study provides evidence that early ethanol deprivation in “high ethanol-drinking” mice leads to a global increase in site-specific editing efficiency of the 5-HT_2C_ receptor and a decrease in the frequency of the less edited D transcript in the hippocampus, suggesting a shift in the 5-HT_2C_ receptor editing pattern towards a lower functioning receptor profile during ethanol abstinence. The observed changes in 5-HT_2C_ receptor editing in the hippocampus were associated with reduced *Tph2* expression in this brain region. We propose that lifelong depletion in the central 5-HT attenuates 5-HT_2C_ receptor editing in the prefrontal cortex and hippocampus.

Limitations to our study include the assessment of only one depression symptom, ‘entrapment’, using the FST screening method. This method may not have been sufficient to detect differences in the aversive aspects of ethanol deprivation between high and low-ethanol drinkers, emphasizing the need for further research in this area. The use of *Tph2*^*−/−*^ mice with complete 5-HT depletion in the brain to verify the impact of reduced brain 5-HT levels may not have been optimal, as it led to more pronounced changes in 5-HT_2C_ receptor editing than ethanol deprivation in wild-type animals. Another weakness of our study are small sample sizes employed for molecular analyses. Nevertheless, they were sufficient to detect significant differences between groups. Additionally, we did not include the ethanol-resistant group in the molecular analyses due to technical limitations related to deep sequencing analysis, which required a high amount of unique primers with different barcodes for each condition (i.e., separate for each animal/treatment/brain region). Furthermore, we conducted our analysis of *Tph2* transcript levels and receptor editing only in the hippocampus and prefrontal cortex and without further subdividing these functionally heterogeneous areas into subregions. Lastly, it is important to note that our results are based on changes in mRNA levels, and we only estimated protein isoforms.

The relevance of these minor alterations in the 5-HT_2C_ receptor mRNA editing on the physiology of 5-HT neurotransmission is unclear and requires further investigation. Therefore, future directions include focusing on wild-type animals and conducting a thorough analysis of metabolic fluctuations in tryptophan (including its conversion to the kynurenine pathway), *Tph2* expression, its enzymatic activity, 5-HT levels, its metabolism, release and reuptake, and 5-HT_2C_ receptor editing during different phases of ethanol consumption, with particular emphasis on early and long-term abstinence (assessment of other depression symptoms), as well as relapse-like behavior. Other goals are to explore whether ethanol deprivation has any effect on the expression of ADAR.

### Supplementary Information

Below is the link to the electronic supplementary material.

Supplementary file1 (PDF 319 KB)

## Data Availability

The datasets generated during and/or analyzed during the current study are available from the corresponding author upon reasonable request.

## Abbreviations

5-HIAA: 5-Hydroxyindoleacetic acid
5-HT: Serotonin
A-to-I: Adenosine-to-inosine RNA editing
ADARs: Adenosine deaminases acting on RNA
ANOVA: Analysis of variance
*C*_T_: Cycle threshold
FST: The forced swim test
INI: The non-edited 5-HT_2C_ receptor isoform
*p*CPA: *para*-Chlorophenylalanine
qPCR: Quantitative real-time PCR
SERT^*−/−*^: Serotonin transporter-deficient mice
SSRIs: Selective serotonin reuptake inhibitors
*Tbp*: TATA box binding protein
TPH1: Tryptophan hydroxylase 1
TPH2: Tryptophan hydroxylase 2
*Tph2*^*−/−*^: *Tph2-*Deficient mice
VGV: The fully-edited 5-HT_2C_ receptor isoform
