# Supplementary material for: Ethanol deprivation and central 5-HT deficiency differentially affect the mRNA editing of the 5-HT2C receptor in the mouse brain
Source: Pharmacol Rep. 2023 Nov 3;75(6):1502–21. doi: 10.1007/s43440-023-00545-6 (PMC10661786; doi:10.1007/s43440-023-00545-6)
Supplement: Supplementary file 1 — Supplementary file1 (PDF 319 KB) [file 43440_2023_545_MOESM1_ESM.pdf]

Ethanol deprivation and central 5-HT deficiency differentially affect the mRNA editing of the 5-HT$_{2C}$ receptor in the mouse brain

Supplementary Results

"High ethanol-drinking" mice exhibit an increased ethanol consumption and preference compared to low ethanol-drinking mice

Ethanol consumption among mice, categorized as high and low-ethanol drinkers, significantly changed over the course of 24 days (a significant ethanol group x day interaction ($F_{23,138}$=1.67, p=0.038, η^2_p=0.22), a significant effect for the ethanol group ($F_{1,6}$=6.35, p=0.045, η^2_p=0.51) and day ($F_{23,138}$=2.17, p=0.0032, η^2_p=0.27); Suppl. Fig. 1a). Post hoc analysis using the Tukey test revealed that high ethanol drinkers exhibited an increased ethanol intake on day 19 compared to day 1 (p=0.004, Suppl. Fig.1a). Ethanol intake levels remained stable in low-ethanol drinkers throughout the 24-day period (p>0.05; Suppl. Fig. 1a).

Ethanol preference was altered by the duration of drinking (a significant ethanol group x day interaction ($F_{23,138}$=1.64, p=0.042, η^2_p=0.22), a significant effect for the ethanol group ($F_{1,6}$=7.08, p=0.037, η^2_p=0.54) and day ($F_{23,138}$=2.05, p=0.006, η^2_p=0.25); Suppl. Fig.1b). The Tukey test demonstrated that high-ethanol drinkers displayed a higher ethanol preference on days 11 and 19 compared to day 1 (p=0.038, and p=0.003, respectively; Suppl. Fig. 1b). There was no change in ethanol preference in the "low ethanol-drinking group" over the 24-day period (p>0.05; Suppl. Fig. 1b).

Supplementary Figures

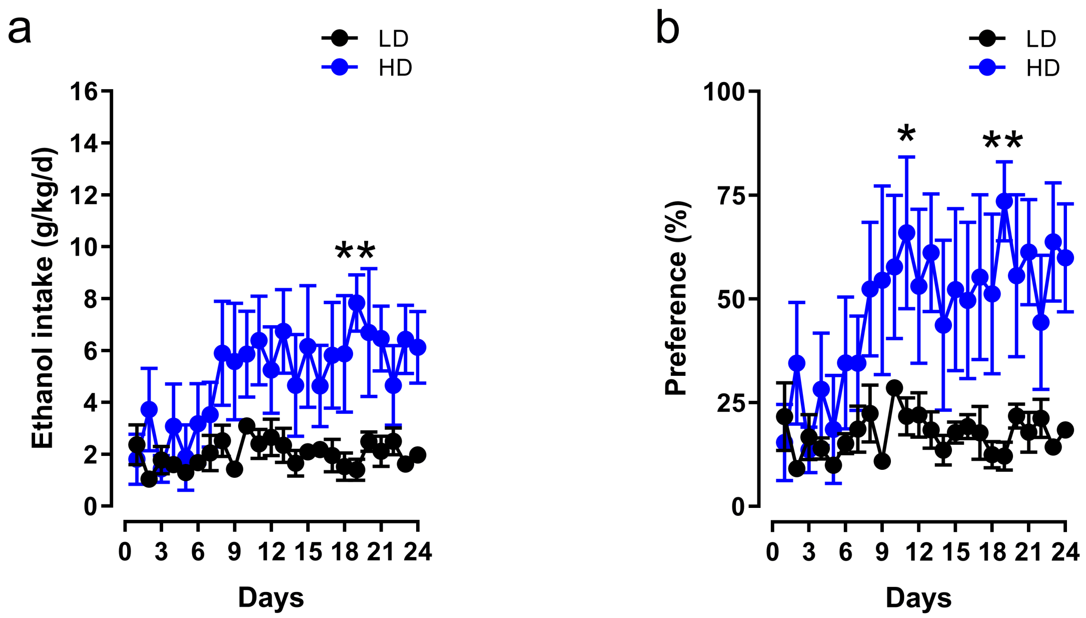

Suppl. Fig. 1 Ethanol consumption and preference in C57BL/6N mice categorized as high and low-ethanol drinkers. During 24 days, the animals had access to both water and 10% ethanol using a two-bottle free-choice procedure. Based on the amount of ethanol consumed during the final week of ethanol drinking, the mice were divided into "high" (HD) and "low (LD) ethanol-drinking" groups. (**a**) ethanol consumption − g of pure ethanol ingested within one day per kg of body weight, (g/kg/d); (**b**) ethanol preference − g of ethanol consumed per day as a percentage of the total liquid consumed. The data is presented as the means (± SEM) (n = 4 mice/group). (**a**) one-way repeated measures ANOVA followed by the Tukey test: $^{**}p<0.01$ vs. day 1 (HD group); $p<0.05$: main effect for the ethanol group; $p<0.01$: main effect for the day. (**b**) one-way repeated measures ANOVA followed by the Tukey test: $^{*}p<0.05$, $^{**}p<0.01$ vs. day 1 (HD group); $p<0.05$: main effect for the ethanol group; $p<0.01$: main effect for the day.
